# Rat-borne diseases at the horizon. A systematic review on infectious agents carried by rats in Europe 1995–2016

**DOI:** 10.1080/20008686.2018.1553461

**Published:** 2019-02-27

**Authors:** Tanja Maria Strand, Åke Lundkvist

**Affiliations:** a Department of Medical Biochemistry and Microbiology, Zoonosis Science Center, Uppsala University, Uppsala, Sweden

**Keywords:** *Capillaria*, Europe, *Hymenolepis*, *Leptospira*, *Rattus*, zoonoses

## Abstract

To investigate the spectrum of rat-borne pathogens circulating in Europe a systematic review spanning across 55 European countries during the years 1995–2016 was performed. The study surveyed viruses, bacteria, macroparasites and unicellular eukaryotes (protozoa). Fifty-three different infectious agents, all with zoonotic potential, were reported to be carried by commensal rats; 48 by the brown rat (*Rattus norvegicus*) and 20 by the black rat (*R. rattus*). There was a tendency for rural areas to harbour more rat-borne microbes than urban areas regarding the brown rat, but the opposite could be observed for the black rat. The study clearly indicated that an improved surveillance on wild rats is needed in Europe, and further indicated the pathogens and geographical areas where the major focus is required. For example, six zoonotic microbes seemed to be clearly more geographically widespread in Europe than others; virulent or resistant *E. coli*, pathogenic *Leptospira* spp., *Hymenolepis diminuta, H. nana, Capillaria hepatica* and *Toxoplasma gondii*.

## Introduction

Several rat-borne zoonotic pathogens and their associated diseases such as hantavirus infections and leptospirosis have recently emerged or re-emerged in Europe (e.g. [1,2]). One pest control company handling approx. 85% of all pest control in Sweden, noted a 67% increase in the number of rat control activities over the last three years (press release 13 February 2017, https://www.anticimex.com/sv-SE/nyhetsrum/2017/saneringar-av-rattor-okar-kraftigt/). The resistance against rodenticides is increasing in rats; the geographic spread of resistance mutations is now well documented in e.g. Great Britain [3]. More rats increase the probability of contact between humans and rats and accordingly the risk for transmission of severe infectious diseases [4].

Brown and black rats (*Rattus norvegicus*, Berkenhout, 1769, and *R. rattus*, Linneaus, 1758) originated in Asia and are to date present all over the globe alongside humans [5–7]. These rodents have introduced pathogenic microorganisms such as e.g. *Yersinia pestis, Bartonella* and hantavirus into many new geographically areas [8–11]. In addition, both black and brown rats have acquired new macroparasites outside their native ranges [12]. Thus, they act as efficient transmitters of pathogens between wildlife, domestic animals, vectors and humans.

The most infamous pathogen known to be transmitted from rodents to humans is *Yersinia pestis*, causing plague. The vectors making the transmission possible are fleas, probably the most effective being the oriental rat flea (*Xenopsylla cheopsis*) [11]. Several mammal species can act as reservoirs for *Yersinia pestis*, but the black rat is a common reservoir in e.g. Madagascar, were over 1500 human plague cases were reported between year 1997 and 2001 [13,14]. Moreover, inhabitants of Madagascar recently suffered from a large plague outbreak with over 2000 reported cases in a period less than three months [15]. Plague is wide-spread and present in large areas of North and South America, Asia and Africa [11].

Lassa virus, which causes a highly fatal hemorrhagic fever common in West Africa, is mainly transmitted directly or indirectly from the multimammate rat (*Mastomys natalensis*) [16–18].

Seoul hantavirus (SEOV), causing severe disease, is mainly found in brown rats in China and Southeast Asia, but also at other locations around the world [10]. The disease caused by SEOV is named haemorrhagic fever with renal syndrome (HFRS) and is characterized mainly by high fever, fatigue and severe kidney problems with a mortality of 2–3% [4].

The biology of the pathogens, hosts and vectors can be altered by urbanization, which may result in an increase of disease transmission [19]. Habitat fragmentation can increase the risk for disease. E.g. in New York, there was a significant increase in the density of *Borrelia burgdorferi* infected tick nymphs, with decreasing forest patch sizes [20]. Globally, 54% of the human population live in urban areas [21]. In Europe, as many as 74% of the approx. 740 million inhabitants (year 2015) live in urban areas.

The major objectives of our study were to:

1. Identify rat-borne pathogens circulating in Europe and reported during the years 1995–2016.
2. Compare pathogen diversity (number of reported pathogenic microorganisms) between different geographical areas/structures of Europe:
  - Compare pathogen abundance in Northern Europe with pathogen abundance in the Southern parts of Europe, and
  - Compare pathogen richness in urban areas with pathogen richness in rural areas and oceanic islands in Europe.

## Materials and methods

Throughout the text, *bacteria* (including rickettsia), *helminths* (including Acanthocephala), unicellular eukaryotes (hereafter called *protozoa* and including also Amoebas and Apicomplexan parasites) and *viruses* were considered. We did not include prions, fungi or ectoparasites (i.e. Arthrodopa) in this study.

## Search strategy

**Figure 1.**
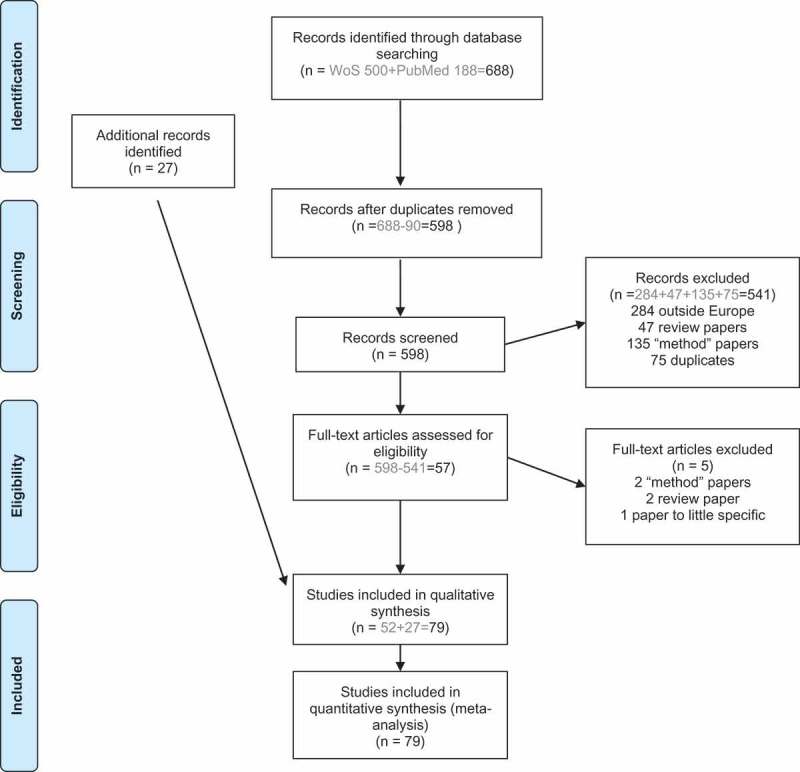
Literature screening for systematic review.

We conducted a systematic literature search to identify articles in the Web of Science ‘All databases’ (including Web of Science Core Collection, BIOSIS Citation Index, BIOSIS Previews, Zoological Records, SciELO Citation Index) and PubMed bibliographic databases as of 26th of September 2016. The time period for articles to be included was set to 1st of January 1995 to the date of search 2016 (26th of September 2016). We followed the search process described in Figure 1.

Text word searches included ‘*Rattus rattus*’, ‘*Rattus norvegicus*’, ‘Norway rat’, ‘brown rat’, ‘black rat’, ‘sewage rat’, ‘urban rat’ or ‘roof rat’) *AND* the **TOPIC**: ‘Rodent-borne’, ‘rat-borne’, Zoono*, ‘human disease’, ‘one health’ or ‘Rat-associated’ or *AND* the **TOPIC**: (*Babesia, Capillaria, Cestodes, Cryptosporidium*, ‘*Echinococcus multiloculari*’, ‘*Entamoeba histolytica’, Fasciolia, Giardia, Hymenolepis, Leishmania*, Nematodes, *Sarcocystis, Taenia*, ‘*Toxocara cati*’, ‘*Toxoplasma gondii*’, Trematodes, Trichinella, *Trypanosoma*, Babesiosis, Capillariasis, Crypdosporidiosis, ‘alveolar echinococcosis’, ‘Amoebic dysentery’, ‘Human fasciolosis’, Giardiasis, Rodentolepiasis, Leishmaniasis, Sarcosporidiosis, Taeniasis, Toxocariasis, Toxoplasmosis, Trichinosis, ‘Chagas disease’, *Anaplasma, Bartonella*, ‘*Borrelia burgdorferi’, Borrelia, Campylobacter*, ‘*Coxiella burnetii*’, ‘*E. coli*’, ‘*Francisella tularensis’, Leptospira, Listeria*, ‘*Orientia tsutsugamushi’, Pasteurella, Pseudomonas, Rickettsia, Salmonella*, ‘*Streptobacillus moniliformis’, Yersinia*, ‘Bartonella Illness’, ‘Lyme disease’, ‘Relapsing fever’, Campylobacteriosis, ‘Q fever’, ‘VTEC’, Tularemia, ‘Leptospirosis’, ‘Weil’s disease’, Listeriosis, ‘Scrub typhus’, Pasterurellosis, Meliodosis, ‘typhus’, ‘murine typhus’, Salmonellosis, ‘rat bite fever’, ‘Haverhill fever’, ‘Yersiniosis’, Plague, Cowpox, ‘Hepatitis E’, HEV, ‘Lymphocytic choriomeningitis’, LCMV, Rabies, ‘Seoul hantavirus’, ‘Seoul virus’, ‘Tick-Borne Encephalitis’, TBE, ‘Haemorrhagic fever’ or ‘Hemorrhagic fever’

The investigation covered the European countries in Table 1 and those were classified in the United Nations division as: *Eastern Europe, Northern Europe, Southern Europe* and *Western Europe* [21].

**Table 1.** Countries included in the literature search.

Western Europe	Southern Europe	Northern Europe	Eastern Europe
Austria	Albania	Denmark	Belarus
Belgium	Andorra	Estonia	Bulgaria
France	Bosnia and Herzegovina	Faeroe Islands	Czech Republic
Germany	Croatia	Finland	Hungary
Liechtenstein	Greece	Iceland	Moldova
Luxembourg	Holy See (Vatican City State)	Ireland	Poland
Monaco	Italy	Latvia	Romania
Netherlands	Malta	Lithuania	Slovakia
Switzerland	Montenegro	Norway	Ukraine
	Portugal	Sweden	
	San Marino	UK	
	Serbia		
	Slovenia		
	Spain		
	TFYR Macedonia		

The Azores, Canary Islands, Faro Islands, Madeira and Svalbard are included in the searches but overseas territories were not included in this study (such as Greenland and Réunion).

The search language was English but articles also in German, French and Italian were included.

## Data abstraction

### Screening

We screened articles that by the title and abstract included the following: a) Brown or black rats, b) Studies performed within the years 1995–2016, and c) Pathogens (harmful or potentially harmful) to humans.

### Exclusion

After the reference program Endnote removed the duplications automatically, 598 papers were present. Articles were excluded if they were performed outside the European countries included in our study (284 papers). Papers that included method descriptions, modelling papers or did not include wild brown or black rats were removed and put in a category named ‘methods’ (135 articles). 47 papers were discarded because they were regarded as reviews. 75 additional duplicated papers were found manually.

Additional articles (n = 27) were added through citation searching.

## Extraction

We extracted the following data from the articles: Type of pathogen (species or at genus level where a genus is known to include species that cause disease in humans, but where no specific species was reported), tissue/tissues sampled, laboratory methods used, rat species, number of rat individuals, number of infested rats, country and habitat. For each rat species and country designation into Eastern, Southern, Northern and Eastern Europe, we recorded and calculated the number of reported pathogens.

Rat studies performed on oceanic islands were designated *island habitat* type no matter if the study was performed in urban or rural environments. Other rat studies (according to the authors) performed in urban, periurban, suburban environment or sewer sites within these habitats were designated *urban habitat*. Studies performed in farms, rural habitats, forests or fields, waste disposal sites and mountain regions were designated *rural habitats*. If it was not possible to find out the habitat type, or if both rural and urban habitat types were mixed, they were not included in the habitat part of the analysis. As for the countries, for each rat species and habitat type, we recorded and calculated the number of pathogens.

## Results

### Literature search

Fifty-seven articles were extracted by the systematic screening for studies on zoonotic rat-borne microbes in Europe and read thoroughly. Four of these 57 papers were excluded since they were found to be solely methodological or reviews. One additional article was removed at this step, a virome study without the requested details presented. A total of 79 articles were finally used, including the additional 27 studies found by the authors.

### Brown and black rats

Five of the 79 articles presented negative results for all rats and pathogens included in the analyses. Sixteen studies on brown rats and five studies on black rats had failed to show any positive results for one or several pathogens.

**Figure 2.**
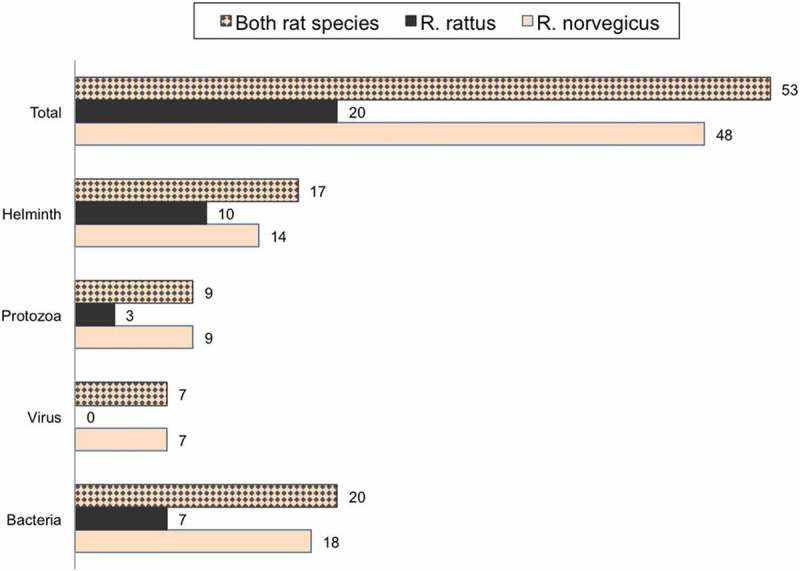
Number of unique pathogens per taxa and rat species. Pathogens combined, regardless of rat species, are also reported.

Among the 74 articles included with at least one positive finding, 55 described pathogens carried by brown rats and only seven studies reported pathogens found in black rats. Twelve studies sampled both brown and black rats for organisms pathogenic for humans. According to our literature search, 53 different infectious agents (or traces of, i.e. agent-specific antibodies) with well-known or potential zoonotic outcome were found in commensal rats in Europe, see Figure 2. The more intensively studied rodent species, the brown rat, was reported to carry 48 unique infectious organisms in total, and the black rat 20 (for details, see Figure 2).

### Geographical distribution of rat-borne pathogens within Europe

Rats from 22 of the 49 European countries and territories included in our initial screening (Table 1, Table S1) have been investigated for at least one rat-borne pathogen between 1995 and 2016.

**Figure 3.**
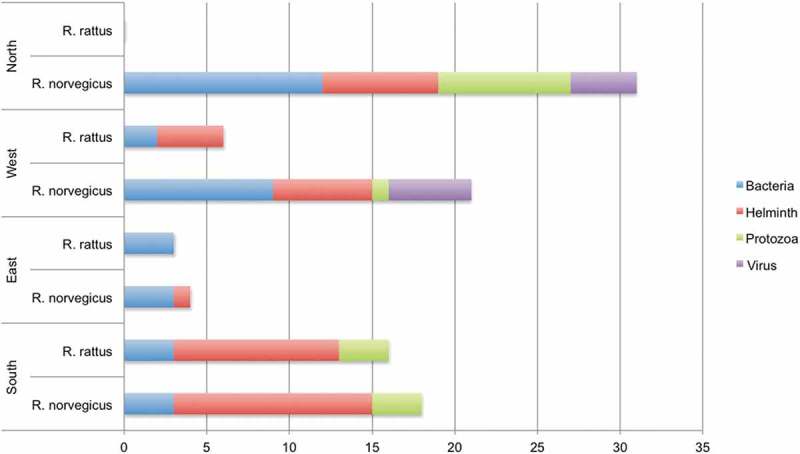
Number of different infectious agents per European zone, rat species and taxonomic level. Where a genus is known to include species that cause disease in humans but no species is given, the genus is counted once for each habitat and rat species.

Regarding the brown rat, most pathogens were described from the Northern zone (Table S1). For the brown rat, rat-borne bacteria and helminths have been detected in all four European zones (Figure 3). No pathogens have been detected in the black rat in the Northern zone (Table S1 and Figure 3). For the black rat, no virus was detected (or tested for) in any of the four zones (Figure 3, Table S1). The studies on the black rat were mainly performed on Oceanic and Mediterranean islands and in countries near the Mediterranean Sea, but also in the Netherlands.

Six infectious organisms were clearly more often reported than others, and they were all present in three geographic zones. These were two bacteria (virulent or resistant *E. coli* and pathogenic *Leptospira* spp.), three helminths (*Hymenolepis diminuta, H. nana* and *Capillaria hepatica*) and one protozoon (*Toxoplasma gondii*).

### Habitat and pathogens

**Figure 4.**
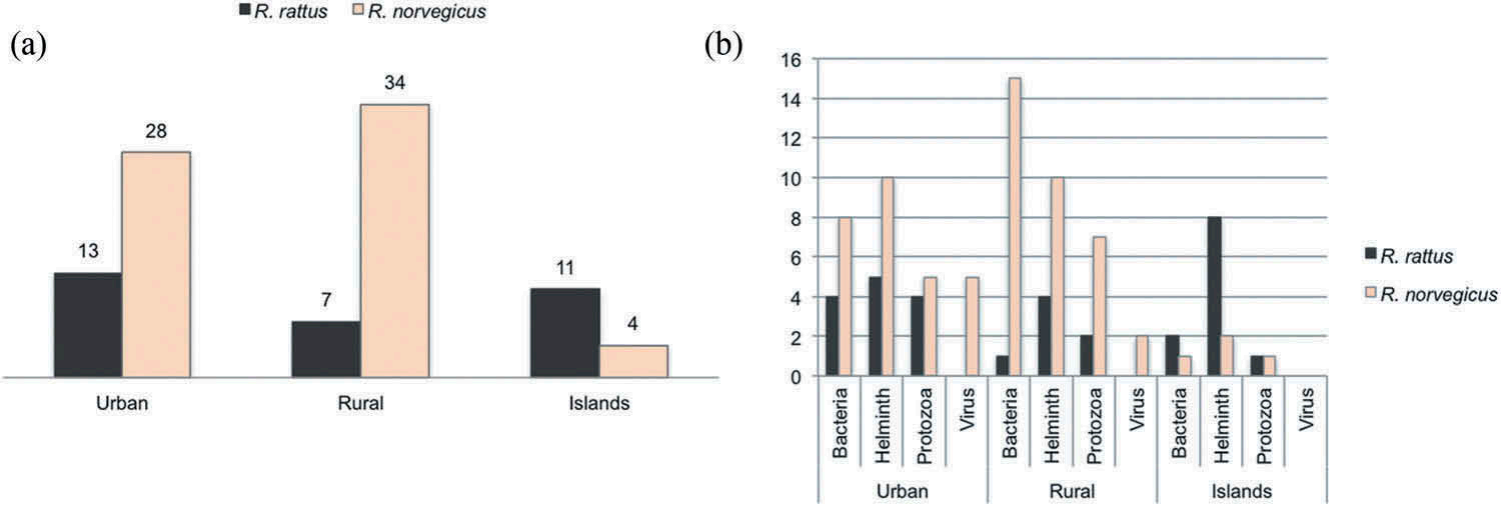
(a) Number of different zoonotic infectious agents found in the literature search by rat species and habitat (**urban**/suburban, **rural**/farm, oceanic **islands)**. Where a genus is known to include species that cause disease in humans but no species is given, the genus is counted once for each habitat and rat species. (b) Number of different zoonotic infectious agents found in the literature search by **taxonomic division**, rat species and habitat.

We compared the number of unique pathogens reported in rats between the specified urban, rural (including studies specified as performed on farms) and oceanic islands habitats (Figure 4(a,b)), most data points were independent studies for each habitat. For the brown rat, rural areas seem to harbour a larger palette of rat-borne organisms than urban areas (Figure 4(a)). The opposite seems to be the case for the black rat (Figure 4(b)). The black rat carried a higher number of pathogens, i. e. helminths, than the brown rat on oceanic islands. Brown rats were free of specific pathogens in three cases of islands habitats, 20 cases of rural environments and 24 cases of urban habitats. Black rats were free of specific pathogens in 10 cases of rural habitats and 11 cases of urban habitats.

## Discussion

### Brown and black rats

In Europe, both the brown and the black rat are present in many geographical areas, although the black rat has disappeared from several of the countries where it used to reside, like e.g. Sweden [22]. In fact, the black rat has been claimed to have been significantly reduced across most of the globe over the last century [23], although exceptions seem to exist [24]. Although this review is not intended as an overview for the various rat species, the brown rat seems to cover the major parts of Europe to date, in contrast to the black rat, which is mainly focused to the warmer coasts and oceanic islands.

Altogether, we found 53 different rat-borne pathogens reported, with potential consequences for the public health in Europe. In an earlier review by Cleaveland and co-workers 180 zoonotic agents that could be hosted by rodents were reported [25]. Globally, there are 164 helminth species reported to be carried by *R. norvegicus*, 156 for *R. rattus*, and only 65 helminths that are common for both *R. rattus* and *R. norvegicus* [12]. In the original distribution areas of the brown and the black rats (Oriental region), they harbour 21 (12.8% of the total global number) and 64 (41% of the total global number) helminths, respectively [12]. According to our review, as many as 17 potentially zoonotic helminths are carried by both rat species. Generally, it is considered that commensal rats can acquire new pathogens alongside their natural pathogens in their new surroundings by so called ‘parasite spillback’ [9,12]. For example, *Coxiella burnetii* in the Netherlands is a zoonotic pathogen that is harboured mainly by goats, sheep and cattle, but was recently found also in rats [26].

### Geographical distribution of rat-borne pathogens within Europe

We suggest a general focus of future surveillance in Europe of the six most widespread pathogens according to this review, i.e. the virulent/resistant *E. coli*, pathogenic *Leptospira* spp., the helminths *Hymenolepis diminuta, H. nana* and *Capillaria hepatica*, and the protozoon *Toxoplasma gondii*.

Humans and rats are like other mammals, natural carriers of the intestinal bacteria *E. coli* [27]. Some variants of *E. coli* can cause direct disease such as for enterohemorrhagic *E. coli* (EHEC/VTEC) that produce verotoxins, or could be harmful once you get another bacterial infection (for antibiotic resistant *E. coli* strains) [28]. Verotoxigenic *E. coli* had year 2015 the highest reported incidence (12.3/100,000) on Ireland and secondly and third in Sweden (5.7/100,000) and in the Netherlands (5.1/100,000), respectively, among the 27 countries in EU reported [29]. Antimicrobial resistance is widespread and is increasing in Europe for *E. coli* as observed by surveillance of human blood or cerebrospinal fluid [30]. A majority of the isolates were resistant to at least one of the antimicrobial groups and many produced Extended-spectrum beta-lactamase (ESBL). The reports of *E. coli* in rats may parallel the status of *E. coli* in humans. Therefore, studies on *E. coli* in rats could be most valuable as an indicator of what strains and what level of antibiotic resistance we actually have in the surroundings. It is not clear whether rats can transmit *E. coli* to the human population but they might serve as excellent mirrors. For example, rats can be monitored in different neighbourhoods and possible be used as an early warning system for resistant types of *E. coli*.

Different *Leptospira* serovars are transmitted by rats and other animals [31]. In most cases leptospirosis in man is rather mild or asymptomatic, but occasionally it may develop to lung, liver and/or kidney disorders, and cardiovascular collapse [32]. Overall, the number of cases of human leptospirosis is generally stable in Europe [33]. Between 2007 and 2015, there were between 439 and 966 reported cases per year, including travel-associated cases [29].

Several European countries or cities have reported increased numbers of domestically acquired leptospirosis during last years. In 2014, the Netherlands experienced an over four-fold increase in the number of autochthonous leptospirosis cases in humans, and also a significant increase of leptospirosis in dogs [1]. Eight of the autochthonous 60 human cases were linked to direct contact with rats. However, several additional cases may have been connected to rats, since the transmission route can also occur indirectly through urine-contaminated water [34]. In addition, the Netherlands reported the highest number of leptospirosis cases in 2015 (including travel-associated disease) (n = 86), alongside Germany (also 86 cases), among the 29 countries reported to ECDC [29]. France does not report a general rise in number of reported leptospirosis cases [33], but in Marseille, the reported incidence of leptospirosis steadily increased from 2001 through 2011, after an absence of cases between 1985–1995 [35]. Marseille is also a town inhabited of many rats and also *Leptospira*-infected rats [33]. In Sweden in 2015, with a recent rise in rat exterminations we detected antibodies against the highly pathogenic *Leptospira* serovar Icterohaemorrhagiae in wild brown rats trapped in Stockholm and Gothenburg [36]. In conclusion, there are strong links established between rats and leptospirosis in humans, also in Europe [34,35]. Since autochthonous human leptospirosis is wide-spread at least in continental Europe [29] we suggest an increased surveillance of pathogenic *Leptospira* in rats, also in areas where *Leptospira* has not (yet) been detected (like Faroe Islands) [37], for an improved awareness of the future threats to the public health.

*Toxoplasma gondii* is a unicellular eukaryote with a life cycle that involves cats [38]. Since rats are living close to humans they have been suggested to be exposed to both cats and *Toxoplasma* oocysts [39]. As shown by this review rats are infected by *T. gondii* all over Europe and they might thereby serve as a good indicator of the spread of this pathogen.

The intestinal tapeworms *Hymenolepis diminuta*, ‘rat tapeworm’, and *H. (*synonym *Rodentolepis) nana*, ‘dwarf tapeworm’ can cause the disease hymenolepiosis but are only occasionally reported from patients in Europe [40,41]. In contrast, our review shows that these parasites are geographically widespread in rats all over Europe. Infection by *H. nana* is one of the most common tapeworm infections in man [42,43] and can also be transmitted from human to human [42]. Both tapeworm species can cause enteritis, although infections often are asymptomatic [44]. *H. nana* has recently been recognised to have zoonotic potential in the UK [45].

Rodents are the main host of the roundworm *Capillaria hepatica* (synonym *Calodium hepaticum*) and among those, the brown rat has the highest prevalence across the globe [46]. The wide distribution in rats is in line with the geographically widespread human hepatic capillariosis in Europe, although a rarely reported disease [47]. It has been suggested that poor and unsanitary conditions together with the presence of rodents and domestic animals increase the risk of infection with *C. hepatica*. Since hepatic capillariosis can be fatal if not diagnosed correctly it is important to know the geographic range of *C. hepatica*.

These three zoonotic helminth parasitic species are all found in rats across a wide geographic distribution in Europe. Still there are not many human cases from Europe reported in the literature. We believe it is important to further survey these helminths to understand their current range and investigate if they are emerging.

We did not find any support for our initial hypothesis that there is a smaller panel of rat-borne pathogens in Northern Europe as compared to more southern areas. In fact, despite the absence of black rats in Fennoscandia [48], we found the majority of the reported rat-borne bacteria in the Northern Europe zone (Figure 3, Table S1). Within Europe, brown rats are more well studied as compared to black rats, they are spread all over Europe and harbour many types of pathogens in large parts of their total geographical range.

### Habitat and pathogens

If there are less species available to serve as reservoirs or carrier-species for pathogens in settled areas, it is possible that at least certain types of pathogens will decrease [19]. One study found that there was a significantly lower total number of zoonotic helminth intensity in rodents in human settlements than in the forests, upland or lowland habitats [49]. On the other hand, if commensal rats are the most competent reservoirs for a certain microbe and are most abundant and other mammal decrease in species and numbers, the microbe may increase in a reverse process of ‘dilution effect’ [50]. Where the rats increase in numbers, the pathogens they harbour may increase too.

Another reason for the possible higher pathogen richness reported from rural areas compared to urban areas in Europe can be that farm areas may have more ‘vacant slots’ for invading brown rats than what is present in towns. Both brown and black rats are thought to not welcome newcomers in already existing populations [5,51]. This may have the effect that exotic rat-borne pathogens do not enter cities as often as they enter rural settings.

One potential limitation of this study is the fact that we were not able to control how publication trends may bias our results. Several factors may influence reporting trends such as varying research fundings and surveillance efforts, which may of course bias the results.

There were not many studies on pathogens in black rats found by our literature screening but the black rat seems to harbour many species of helminths, especially on the Oceanic islands (Figure 4(b)). We therefore have to reject our hypothesis about a smaller panel of pathogens in oceanic islands than other areas (urban and rural). Both rat species colonised the world at different routes and periods [5,6,23,52]. The black rat is generally thought to have been established in Europe earlier than the brown rat [23,52]. Molecular results indicate that the black rat has a long colonisation history on the Canary Islands and at least the helminths pathogenic to man may have had a long time to co-evolve with the black rat.

We recommend future studies to include and present data both from rural and urban surveys of rat-borne pathogens in the same article to make comparisons possible. For surveillance, we recommend a sample size calculated from an expected prevalence.

## Conclusion

These results will ultimately serve as guidelines for future monitoring of rat-borne zoonoses in areas where data is lacking.

Our results also identified a number of important human pathogens that are carried by increasing populations of rats, and thereby may constitute significant and/or increasing threats to the Public Health. We suggest that wild rats in Europe are monitored regularly at least for the six most widespread pathogens identified, i.e. virulent/resistant *E. coli*, pathogenic *Leptospira* spp., the helminths *Hymenolepis diminuta, H. nana* and *Capillaria hepatica*, and the protozoon *Toxoplasma gondii*.
