# Supplementary material for: Rat-borne diseases at the horizon. A systematic review on infectious agents carried by rats in Europe 1995–2016
Source: Infect Ecol Epidemiol. 2019 Feb 27;9(1):1553461. doi: 10.1080/20008686.2018.1553461 (PMC6394330; doi:10.1080/20008686.2018.1553461)
Supplement: Supplemental Material [file ZIEE_A_1553461_SM7499.docx]

| **Taxonomic level** | **Species or genus** | **Northern** | | **Eastern** | | **Western** | | **Southern** | |  |
| --- | --- | --- | --- | --- | --- | --- | --- | --- | --- | --- |
| **Subgroup** | **Infectious organism**  **(no. of unique pathogen per taxa)** | ***R. norvegicus*** | ***R. rattus*** | ***R. norvegicus*** | ***R. rattus*** | ***R. norvegicus*** | ***R. rattus*** | ***R. norvegicus*** | ***R. rattus*** | ***References*** |
|  |  |  |  |  |  |  |  |  |  |  |
| **Family** | **Bacteria (20)** |  |  |  |  |  |  |  |  |  |
| *Anaplasmataceae* | *Anaplasma phagocytophilum* |  |  |  | 1 |  |  |  |  | [1] |
| *Bartonellaceae* | *Bartonella* spp. |  |  |  |  |  |  | 1 | 1 | [2] |
|  | *Bartonella tribocorum* |  |  |  |  | 1 |  | 1 |  | [3, 4] |
| *Brachyspiraceae* | *Brachyspira pilosicoli +* spp.' | 1 |  |  |  |  |  |  |  | [5] |
| *Campylobacteraceae* | *Campylobacter coli* | 1 |  |  |  |  |  |  |  | [6] |
|  | *Campylobacter jejuni* | 1 |  |  |  |  |  |  |  | [6] |
| *Coxiellaceae* | *Coxiella burnetii* | 1 |  |  |  | 2 | 1 |  |  | [7-10] |
| *Enterobacteriaceae* | *E. coli** | 1 |  | 1 |  | 1 |  |  |  | [11-14] |
|  | *Salmonella enterica* | 1 |  |  |  |  |  |  |  | [15] |
|  | *Salmonella* spp. |  |  |  |  | 1 |  |  |  | [10] |
|  | *Yersinia enterocolitica* | 2 |  |  |  |  |  |  |  | [7, 16, 17] |
|  | *Yersinia* spp. |  |  |  |  | 1 |  |  |  | [10] |
| *Francisellaceae* | *Francisella tularensis* |  |  |  | 1 |  |  |  | 1 | [1, 18] |
| *Leptospiraceae* | *Leptospira* spp.*** | 3 |  |  |  | 3 |  | 2 | 2 | [6, 7, 10, 19-29] |
| *Listeriaceae* | *Listeria* spp. | 1 |  |  |  |  |  |  |  | [7] |
| *Mycoplasmataceae* | *Mycoplasma* spp. |  |  | 1 |  |  |  |  |  | [30] |
| *Pasteurellaceae* | *Pasteurella* spp. | 1 |  |  |  |  |  |  |  | [7] |
| *Pseudomonadaceae* | *Pseudomonas* spp. | 1 |  |  |  |  |  |  |  | [7] |
| *Rickettsiaceae* | *Rickettsia helvetica* + spp.' |  |  |  |  | 1 |  |  |  | [29] |
|  | *Rickettsia typhi* |  |  |  |  |  |  | 1 |  | [31] |
| *Spirochaetaceae* | *Borrelia burgdorferi* |  |  | 1 | 1 | 1 |  |  |  | [1, 30, 32] |
| *Staphylococcaceae* | *Staphylococcus aureus** |  |  |  |  | 1 | 1 |  |  | [33] |
| *Vibrionaceae* | *Vibrio* sp. | 1 |  |  |  |  |  |  |  | [16] |
|  |  |  |  |  |  |  |  |  |  |  |
| **Subgroup** | **Helminths (17)** |  |  |  |  |  |  |  |  |  |
| Acanthocephala |  |  |  |  |  |  |  |  |  |  |
| *Acanthocephala* | *Acanthocephala* spp. |  |  |  |  |  |  | 1 | 1 | [34, 35] |
| Cestoda |  |  |  |  |  |  |  |  |  |  |
| *Hymenolepidade* | *Hymenolepis diminuta* | 1 |  |  |  | 1 | 1 | 2 | 3 | [7, 34-39] |
|  | *Hymenolepis* spp. | 1 |  |  |  |  |  |  |  | [16] |
|  | *Hymenolepis** fraterna* |  |  |  |  | 1 | 1 | 1 | 1 | [38,39] |
|  | *Hymenolepis** microstoma* |  |  |  |  |  |  |  | 1 | [38] |
|  | *Hymenolepis** nana* | 1 |  |  |  | 1 |  | 1 | 1 | [7, 34, 35, 39, 40] |
| *Taeniidae* | *Taenia taeniaeformis* | 1 |  |  |  |  |  | 2 |  | [7, 35, 41] |
| Nematoda |  |  |  |  |  |  |  |  |  |  |
| *Ascaridoidea* | *Ascaris* spp. |  |  |  |  | 1 |  |  |  | [42] |
|  | *Toxocara cati* | 1 |  |  |  |  |  |  |  | [7] |
|  | *Toxocara* spp*.* | 1 |  |  |  |  |  | 1 |  | [16, 42] |
| *Plagiorchiidae* | *Plagiorchis muris* |  |  |  |  | 1 |  | 1 |  | [39] |
| *Spiruridea* | *Gongylonema neoplasticum* |  |  |  |  |  |  |  | 1 | [36] |
|  | *Gongylonema* spp. |  |  |  |  |  |  | 1 |  | [35] |
| *Thricinelloidea* | *Capillaria hepatica**** + spp.' | 1 |  |  |  | 1 | 1 | 4 | 2 | [7, 16, 34-37, 40-46] |
|  | *Capillaria**** annulosa* |  |  |  |  |  |  |  | 1 | [36] |
|  | *Trichinella britovi* |  |  |  |  |  |  | 1 | 1 | [47] |
|  | *Trichinella spiralis* | 1 |  |  |  |  |  | 1 |  | [48, 49] |
|  | *Trichinella* sp. | 1 |  | 1 |  |  |  |  |  | [48, 50] |
|  | *Trichuris* sp. | 1 |  |  |  |  |  |  |  | [16] |
| Trematoda |  |  |  |  |  |  |  |  |  |  |
| Fasciolidae | *Fasciola hepatica* |  |  |  |  |  | 1 |  |  | [51] |
| Brachylaimidae | *Brachylaima* spp. |  |  |  |  |  |  | 1 | 2 | [34-36] |
|  |  |  |  |  |  |  |  |  |  |  |
| **Subgroup** | **Protozoa (9 )** |  |  |  |  |  |  |  |  |  |
| *Cryptosporidiidae* | *Cryptosporidium muris* + spp.' | 1 |  |  |  |  |  |  |  | [6] |
| *Entamoebidae* | *Cryptosporidium parvum* | 1 |  |  |  |  |  |  | 1 | [7, 52, 53] |
| *Hexamitidae* | *Entamoeba* spp. | 1 |  |  |  |  |  |  |  | [16] |
|  | *Eimeria* spp. | 1 |  |  |  |  |  |  |  | [7] |
| *Sarcocystidae* | *Giardia intestinalis* | 1 |  |  |  |  |  | 1 | 1 | [6, 54] |
| *Sarcocystidae* | *Toxoplasma gondii* | 1 |  |  |  | 1 |  | 1 |  | [7, 16, 26, 55, 56] |
| *Trypanosomatidae* | *Neospora caninum* | 1 |  |  |  |  |  |  |  | [56] |
|  | *Trypanosoma lewisi* | 1 |  |  |  |  |  |  |  | [7] |
|  | *Leishmania infantum* + spp.' |  |  |  |  |  |  | 2 | 1 | [57-59] |
|  |  |  |  |  |  |  |  |  |  |  |
| **Family** | **Virus (7)** |  |  |  |  |  |  |  |  |  |
| **ssRNA±** |  |  |  |  |  |  |  |  |  |  |
| *Bunyaviridae* | *Seoul hantavirus****** | 1 |  |  |  | 3 |  |  |  | [7, 26, 60-63] |
| *Caliciviridae* | *Norovirus* | 1 |  |  |  |  |  |  |  | [64] |
|  | *Sapovirus* |  |  |  |  | 1 |  |  |  | [65] |
| *Hepeviridae* | *Hepatitis E* | 1 |  |  |  | 2 |  |  |  | [26, 64, 66-69] |
| *Picornaviridae* | *Encephalomyocarditis virus* | 1 |  |  |  |  |  |  |  | [6] |
| **dsRNA** |  |  |  |  |  |  |  |  |  |  |
| *Reoviridae* | *Rotavirus A* |  |  |  |  | 1 |  |  |  | [65] |
| **dsDNA** |  |  |  |  |  |  |  |  |  |  |
| *Poxviridae* | *Cowpox and Orthopoxvirus* |  |  |  |  | 2 |  |  |  | [29, 70, 71] |

| 'Undiagnosed species but sampled in the same countries as the described |
| --- |
| *pathogenic/resistant/virulent |
| ** synonym *Rodentolepis* |
| *** synonym *Calodium hepaticum* |
| **** synonym *Aonchotheca* |
| *****described also as Hantavirus/Hantaan virus in the articles |

**References**

(1) **Christova I, Gladnishka T.** Prevalence of infection with *Francisella tularensis*, *Borrelia burgdorferi* sensu lato and *Anaplasma phagocytophilum* in rodents from an endemic focus of tularemia in Bulgaria. *Annals of agricultural and environmental medicine* 2005; **12**(1): 149-152.

(2) **Ellis BA, et al.** Rats of the genus *Rattus* are reservoir hosts for pathogenic *Bartonella* species: an Old World origin for a New World disease? *The Journal of infectious diseases* 1999; **180**(1): 220-224.

(3) **Marquez FJ, et al.** Molecular screening of *Bartonella* species in rodents from South Western Spain. *Vector-Borne and Zoonotic Diseases* 2008; **8**(5): 695-700.

(4) **Heller R, et al.** *Bartonella tribocorum* sp. nov., a new *Bartonella* species isolated from the blood of wild rats. *International Journal of Systematic Bacteriology* 1998; **48**: 1333-1339.

(5) **Backhans A, et al.** Typing of *Brachyspira* spp. from rodents, pigs and chickens on Swedish farms. *Veterinary microbiology* 2011; **153**(1): 156-162.

(6) **Backhans A, et al.** Occurrence of pathogens in wild rodents caught on Swedish pig and chicken farms. *Epidemiology and infection* 2012; **1**(1): 1-7.

(7) **Webster JP, Macdonald DW.** Parasites of wild brown rats (*Rattus norvegicus*) on UK farms. *Parasitology* 1995; **111**: 247-255.

(8) **Webster JP, Lloyd G, Macdonald DW.** Q fever (*Coxiella burnetii*) reservoir in wild brown rat (*Rattus norvegicus*) populations in the UK. *Parasitology* 1995; **110 ( Pt 1)**: 31-35.

(9) **Reusken C, et al.** *Coxiella burnetii* (Q fever) in *Rattus norvegicus* and *Rattus rattus* at livestock farms and urban locations in the Netherlands; could *Rattus* spp. represent reservoirs for (re)introduction? *Preventive veterinary medicine* 2011; **101**(1-2): 124-130.

(10) **Runge M, et al.** Distribution of rodenticide resistance and zoonotic pathogens in Norway rats in Lower Saxony and Hamburg, Germany. *Pest Management Science* 2013; **69**(3): 403-408.

(11) **Čížek A, et al.** Shiga toxin-producing *Escherichia coli* O157 in feedlot cattle and Norwegian rats from a large-scale farm. *Letters in Applied Microbiology* 1999; **28**(6): 435-439.

(12) **Nielsen EM, et al.** Verocytotoxin-producing *Escherichia coli* in wild birds and rodents in close proximity to farms. *Applied and environmental microbiology* 2004; **70**(11): 6944-6947.

(13) **Guenther S, et al.** Detection of pandemic B2-O25-ST131 *Escherichia coli* harbouring the CTX-M-9 extended-spectrum beta-lactamase type in a feral urban brown rat (*Rattus norvegicus*). *Journal of Antimicrobial Chemotherapy* 2010; **65**(3): 582-584.

(14) **Guenther S, et al.** Frequent combination of antimicrobial multiresistance and extraintestinal pathogenicity in *Escherichia coli* isolates from urban rats (*Rattus norvegicus*) in Berlin, Germany. 2012; **7**(11).

(15) **Hilton AC, Willis RJ, Hickie SJ.** Isolation of *Salmonella* from urban wild brown rats (*Rattus norvegicus*) in the West Midlands, UK. *International journal of environmental health research* 2002; **12**(2): 163-168.

(16) **Battersby SA, Parsons R, Webster JP.** Urban rat infestations and the risk to public health. *Journal of Environmental Health Research* 2002; **1**: 57-65.

(17) **Backhans A, Fellstrom C, Lambertz ST.** Occurrence of pathogenic *Yersinia enterocolitica* and *Yersinia pseudotuberculosis* in small wild rodents. *Epidemiology and infection* 2011; **139**(8): 1230-1238.

(18) **Reintjes R, et al.** Tularemia outbreak investigation in Kosovo: case control and environmental studies. *Emerging infectious diseases* 2002; **8**(1): 69-73.

(19) **Brem S, et al.** *Leptospira* infected rat population probably causing a case of fatal morbus Weil. *Berliner Und Munchener Tierarztliche Wochenschrift* 1995; **108**(11): 405-407.

(20) **Webster JP, Ellis WA, MacDonald DW.** Prevalence of *Leptospira* and other zoonoses in wild brown rats on UK farms. *Mammalia* 1995; **59**(4): 615-622.

(21) **Amaddeo D, et al.** Leptospirosis in wild rodents living in urban areas (Rome-Italy). *Proccedings of the I European Congress of Mammalogy*, 1996, pp. 105-114.

(22) **Collares-Pereira M, et al.** First epidemiological data on pathogenic leptospires isolated on the Azorean islands. *European Journal of Epidemiology* 1997; **13**(4): 435-441.

(23) **Pezzella M, et al.** Leptospirosis survey in wild rodents living in urban areas of Rome. *Annali di igiene : medicina preventiva e di comunita* 2004; **16**(6): 721-726.

(24) **Aviat F, et al.** *Leptospira* exposure in the human environment in France: A survey in feral rodents and in fresh water. *Comparative immunology, microbiology and infectious diseases* 2009; **32**(6): 463-476.

(25) **Socolovschi C, et al.** Strikes, flooding, rats, and leptospirosis in Marseille, France. *International journal of infectious diseases* 2011; **15**(10): e710-e715.

(26) **Ayral F, et al.** The relationship between socioeconomic indices and potentially zoonotic pathogens carried by wild Norway rats: a survey in Rhone, France (2010-2012). *Epidemiology and infection* 2015; **143**(3): 586-599.

(27) **Ayral F, et al.** Distribution of *Leptospira interrogans* by Multispacer Sequence Typing in urban Norway rats (*Rattus norvegicus*): a survey in France in 2011-2013. *PloS one* 2015; **10**(10).

(28) **Strand TM, et al.** Highly pathogenic *Leptospira* found in urban brown rats (*Rattus norvegicus*) in the largest cities of Sweden. *Vector-Borne and Zoonotic Diseases* 2015; **15**(12): 779-781.

(29) **Heuser E, et al.** Survey for zoonotic pathogens in Norway rat populations from Europe. *Pest management science* 2016.

(30) **Hornok S, et al.** Synanthropic rodents and their ectoparasites as carriers of a novel haemoplasma and vector-borne, zoonotic pathogens indoors. *Parasites & Vectors* 2015; **8**.

(31) **Tselentis Y, et al.** Genotypic identification of murine typhus *Rickettsia* in rats and their fleas in an endemic area of Greece by the polymerase chain reaction and restriction fragment length polymorphism. *American Journal of Tropical Medicine and Hygiene* 1996; **54**(4): 413-417.

(32) **Matuschka FR, et al.** Risk of urban lyme disease enhanced by the presence of rats. *The Journal of infectious diseases* 1996; **174**(5): 1108-1111.

(33) **Van de Giessen A, et al.** Occurrence of methicillin-resistant *Staphylococcus aureus* in rats living on pig farms. *Preventive veterinary medicine* 2009; **91**(2): 270-273.

(34) **Milazzo C, et al.** Helminth fauna of commensal rodents, *Mus musculus* (Linnaeus, 1758) and *Rattus rattus* (Linnaeus, 1758)(*Rodentia, Muridae*) in Sicily (Italy). *Revista Ibero-Latinoamericana de Parasitología* 2010; **69**(2): 194-198.

(35) **Milazzo C, et al.** Helminths of the brown rat (*Rattus norvegicus*)(Berkenhout, 1769) in the city of Palermo, Italy. *Helminthologia* 2010; **47**(4): 238-240.

(36) **Casanova J, et al.** On the helminthofauna of wild mammals (*Rodentia*, *Insectivora* and *Lagomorpha*) in Azores archipelago (Portugal). *Vie et milieu* 1996; **46**(3-4): 253-259.

(37) **Milazzo C, et al.** Helminths and ectoparasites of *Rattus rattus* and *Mus musculus* from Sicily, Italy. *Comparative Parasitology* 2003; **70**(2): 199-204.

(38) **Foronda P, et al.** Distribution and genetic variation of hymenolepidid cestodes in murid rodents on the Canary Islands (Spain). *Parasites & Vectors* 2011; **4**.

(39) **Franssen F, et al.** Helminth parasites in black rats (*Rattus rattus*) and brown rats (*Rattus norvegicus*) from different environments in the Netherlands. *Infection ecology & epidemiology* 2016; **6**: 31413-31413.

(40) **McGarry JW, et al.** Zoonotic helminths of urban brown rats (*Rattus norvegicus*) in the UK: Neglected Public Health Considerations? *Zoonoses and public health* 2015; **62**(1): 44-52.

(41) **Kataranovski M, et al.** First record of *Calodium hepaticum* and *Taenia taeniaeformis* liver infection in wild Norway rats (*Rattus norvegicus*) in Serbia. *Archives of Biological Sciences* 2010; **62**(2): 431-440.

(42) **Stojcevic D, Marinculic A, Mihaljevic Z.** Prevalence of *Capillaria hepatica* in Norway rats (*Rattus norvegicus*) in Croatia. *Veterinarski Arhiv* 2002; **72**(3): 141-149.

(43) **Davoust B, et al.** Research for three parasitic infections within a population of rats trapped in Marseille: Evaluation of the zoonotic risk. *Bulletin De L Academie Nationale De Medecine* 1997; **181**(5): 887-897.

(44) **Ceruti R, et al.** *Capillaria hepatica* infection in wild brown rats (*Rattus norvegicus*) from the urban area of Milan, Italy. *Journal of Veterinary Medicine Series B-Infectious Diseases and Veterinary Public Health* 2001; **48**(3): 235-240.

(45) **Millan J, et al.** Factors associated with the prevalence and pathology of *Calodium hepaticum* and *C. splenaecum* in periurban micromammals. *Parasitology research* 2014; **113**(8): 3001-3006.

(46) **Redrobe SP, Patterson-Kane JC.** *Calodium hepaticum* (syn. *Capillaria hepatica*) in captive rodents in a zoological garden. *Journal of Comparative Pathology* 2005; **133**(1): 73-76.

(47) **Pozio E, et al.** Environmental and human influence on the ecology of *Trichinella spiralis* and *Trichinella britovi* in Western Europe. *Parasitology* 1996; **113**(06): 527-533.

(48) **Mikkonen T, et al.** Spatial variation of *Trichinella* prevalence in rats in Finnish waste disposal sites. *The Journal of parasitology* 2005; **91**(1): 210-213.

(49) **Stojcevic D, et al.** The epidemiological investigation of *Trichinella* infection in brown rats (R*attus norvegicus*) and domestic pigs in Croatia suggests that rats are not a reservoir at the farm level. *The Journal of parasitology* 2004; **90**(3): 666-670.

(50) **Hurníková Z, et al.** First record of *Trichinella pseudospiralis* in the Slovak Republic found in domestic focus. *Veterinary parasitology* 2005; **128**(1–2): 91-98.

(51) **Valero MA, et al.** Patterns in size and shedding of *Fasciola hepatica* eggs by naturally and experimentally infected murid rodents. *Journal of Parasitology* 2002; **88**(2): 308-313.

(52) **Quy RJ, et al.** The Norway rat as a reservoir host of *Cryptosporidium parvum*. *J Wildlife Dis* 1999; **35**(4): 660-670.

(53) **Webster JP, Macdonald DW.** Cryptosporidiosis reservoir in wild brown rats (*Rattus norvegicus*) in the UK. *Epidemiology & Infection* 1995; **115**(1): 207-209.

(54) **Fernandez-Alvarez A, et al.** Identification of a novel assemblage G subgenotype and a zoonotic assemblage B in rodent isolates of *Giardia duodenalis* in the Canary Islands, Spain. *Parasitology* 2014; **141**(2): 206-215.

(55) **Gotteland C, et al.** Species or local environment, what determines the infection of rodents by *Toxoplasma gondii*? *Parasitology* 2014; **141**(2): 259-268.

(56) **Hughes J, et al.** The prevalence of *Neospora caninum* and co-infection with *Toxoplasma gondii* by PCR analysis in naturally occurring mammal populations. *Parasitology* 2006; **132**(01): 29-36.

(57) **Papadogiannakis E, et al.** Molecular detection of *Leishmania infantum* in wild rodents (*Rattus norvegicus*) in Greece. *Zoonoses Public Health* 2010; **57**(7‐8): e23-e25.

(58) **Helhazar M, et al.** Natural infection of synathropic rodent species *Mus musculus* and *Rattus norvegicus* by *Leishmania infantum* in Sesimbra and Sintra - Portugal. *Parasites & Vectors* 2013; **6**.

(59) **Di Bella C, et al.** Are rodents a potential reservoir for *Leishmania infantum* in Italy? *Ibex Journal of Mountain Studies* 2003; **7**: 125-129.

(60) **Heyman P, et al.** Serological and genetic evidence for the presence of Seoul hantavirus in *Rattus norvegicus* in Flanders, Belgium. *Scandinavian journal of infectious diseases* 2009; **41**(1): 51-56.

(61) **Jameson L, et al.** The continued emergence of hantaviruses: isolation of a Seoul virus implicated in human disease, United Kingdom, October 2012. *Euro surveillance : bulletin Europeen sur les maladies transmissibles = European communicable disease bulletin* 2013; **18**(1): 4-7.

(62) **McCaughey C, et al.** Evidence of hantavirus in wild rodents in Northern Ireland. *Epidemiology & Infection* 1996; **117**(02): 361-366.

(63) **Verner-Carlsson J, et al.** First evidence of Seoul hantavirus in the wild rat population in the Netherlands. *Infection ecology & epidemiology* 2015; **5**: 27215-27215.

(64) **Wolf S, et al.** The simultaneous occurrence of human norovirus and hepatitis E virus in a Norway rat (*Rattus norvegicus*). *Arch Virol* 2013; **158**(7): 1575-1578.

(65) **Sachsenroeder J, et al.** Metagenomic identification of novel enteric viruses in urban wild rats and genome characterization of a group A rotavirus. *Journal of General Virology* 2014; **95**: 2734-2747.

(66) **Widen F, et al.** PCR detection and analyzis of potentially zoonotic Hepatitis E virus in French rats. *Virology Journal* 2014; **11**.

(67) **Johne R, et al.** Rat hepatitis E virus: Geographical clustering within Germany and serological detection in wild Norway rats (*Rattus norvegicus*). *Infection Genetics and Evolution* 2012; **12**(5): 947-956.

(68) **Johne R, et al.** Detection of a novel hepatitis E-like virus in faeces of wild rats using a nested broad-spectrum RT-PCR. *Journal of General Virology* 2010; **91**: 750-758.

(69) **Johne R, et al.** Novel Hepatitis E virus genotype in Norway rats, Germany. *Emerging Infectious Diseases* 2010; **16**(9): 1452-1455.

(70) **Kurth A, et al.** Rat-to-elephant-to-human transmission of cowpox virus. *Emerging Infectious Diseases* 2008; **14**(4): 670-671.

(71) **Wolfs TFW, et al.** Rat-to-human transmission of cowpox infection. *Emerging Infectious Diseases* 2002; **8**(12): 1495-1496.
